# Failure Analysis of Cement Sheath Mechanical Integrity Based on the Statistical Damage Variable

**DOI:** 10.1021/acsomega.2c06164

**Published:** 2023-01-06

**Authors:** Xuning Wu, Jian Liu, Zaoyuan Li, Weitao Song, Yang Liu, Qing Shi, Rui Chen

**Affiliations:** † State Key Laboratory of Oil and Gas Reservoir Geology and Exploitation, Southwest Petroleum University, Chengdu610500, China; ‡ CCDC Downhole Operation Company, Chengdu610052, China

## Abstract

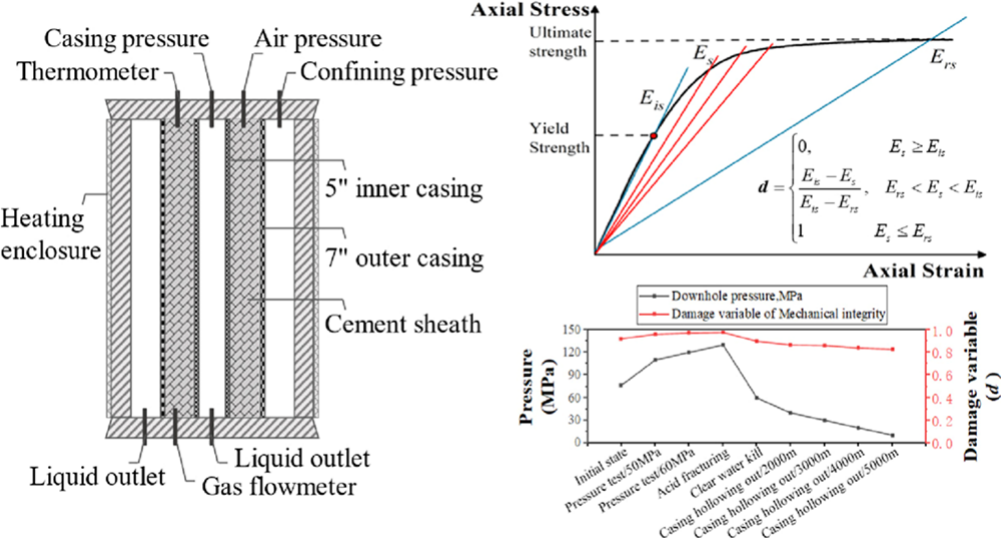

Maintaining the cement sheath mechanical integrity is the key to ensuring the benefit and safety of oil and gas well drilling and production. The main function of the cement sheath is to isolate oil and gas from water layers, which prevents the formation fluid from channeling to other layers. At present, how to effectively evaluate the cement sheath sealing performance is the fundamental problem to be solved urgently. This paper first carried out the cement sheath annulus isolation experiment and analyzed the main forms of cement sheath seal failure. Then, the interaction of the cement sheath, casing, and surrounding rock in the initial state and subsequent wellbore operations was explained. An analysis model for the cement sheath mechanical integrity incorporating the nonlinear elastic constitutive equation was proposed. Based on the statistical damage variable in continuum mechanics theory, a damage variable was established. The results show that the main form of cement sheath integrity failure is tensile crack damage and micro-annulus caused by plastic yielding. The damage variable can quantitatively describe the cement sheath mechanical integrity. The field case analysis shows that the damage variable is highly correlated with wellbore pressure and also verifies the applicability of the variable. Reducing wellbore pressure will help maintain the mechanical integrity of the cement sheath, providing sealing performance. This research can provide a reference for designing the mechanical properties of the cement sheath and improving the sealing ability of the cement sheath.

## 1 Introduction

Portland cement stone is a brittle material with congenital defects. During the drilling and production of oil and gas wells, under the action of the surrounding rock stress and wellbore stress,^1,2^ the cement sheath is easily broken to form macroscopic cracks, which become gas channeling paths and cause sustained casing pressure.^3−5^ Maintaining the sealing integrity of the cement sheath is the key to ensuring the long-term safe production of oil and gas wells, and how effectively evaluating the sealing performance of the cement sheath is a basic problem that needs to be solved urgently.^6^ At present, the direct way is to evaluate the damage and failure mode of the cement sheath through annulus isolation simulation experiments.

The general practice^7−10^ of the annulus isolation simulation experiment is to use full-size casing to form an inner and outer cylinder and inject cement slurry between the inner and outer cylinders to become a cement sheath. The sealing performance of cement sheath is investigated by simulating the pressure change by pressurizing or depressurizing inside the inner barrel, or simulating the downhole temperature change by heating and cooling inside the inner barrel. The application of the cement sheath seal integrity evaluation device intuitively shows the failure form of the cement sheath, and the established devices can be divided into full-scale devices and non-full-scale devices. The design of the full-scale device is mainly based on the size of the casing and the cement sheath in the cementing interval of the oil layer, while the non-full-scale device adopts the equivalent stress theory to restore the stress environment of the cement sheath in the downhole.

In 1992, Goodwin and Crook^7^ established a simulated wellbore with two layers of casing inside and outside, and a cement sheath was formed between the two layers of casing by grouting and curing. The effect of the stress–strain process of the casing and the cement sheath on the sealing ability of the assembly was tested by gradually increasing the casing pressure. The experiment results prove that tensile crack damage is one of the main forms of cement sheath integrity failure. In 1993, Jackson and Murphey^8^ also produced a similar simulation device by gradually increasing the casing pressure and then depressurizing it to a stable value to test the sealing ability of the cement sheath under cyclic loading and unloading conditions. The experiment results prove that plastic yielding is one of the main ways of cement sheath sealing failure. Physical experiments by Boulkhelifa et al.,^9^ Li et al.,^11^ and numerical simulations by Zhou et al.^12^ also demonstrated tensile crack damage and plastic yielding of cement sheath.

From previous research, it can be concluded that the main form of cement sheath integrity failure is tensile crack damage and micro-annulus caused by plastic yielding. The long cycle and high cost of the annulus isolation experiment lead to limitations in field applications. Therefore, in this research, aiming at the failure mechanism of cement sheath integrity, laboratory experiments are carried out to test the failure modes of cement sheath. Then, based on the continuum mechanics theory, the damage variable is established. Finally, the field application is used as the inspection standard to achieve the purpose of effectively evaluating the cement sheath integrity.

## 2 Experiment Materials and Methods

### 2.1 Cement Slurry System

Two kinds of cement slurry systems with the same density are mainly tested, one of which is an elastic cement slurry system. The detailed ingredient is as follows:

(1) 1.90 g/cm^3^ elastic cement (type B): Grade G cement + flexible anti-channeling agent + fiber + dispersant + fluid loss reducer + retarder + defoamer + water (W/S = 0.503), referred to as B190;
(2) 1.90 g/cm^3^ pure cement: Grade G cement + dispersant + stabilizer + fluid loss agent + defoamer + water (W/S = 0.38), referred to as Y190.

The curing conditions of the cement slurry are room temperature (20 °C) and pressure (0.1 MPa), and time of 7 days. The basic physical properties of the two types of cement stone are shown in Table 1.

**Table 1:** Basic Physical Properties of Cement Stone

cement stone	compressive strength (MPa)	Young’s modulus (GPa)	Poisson ratio
B190	24.70	6.50	0.27
Y190 (pure cement)	31.01	6.25	0.35

### 2.2 Annular Isolation Simulation Device

**Figure 1:**
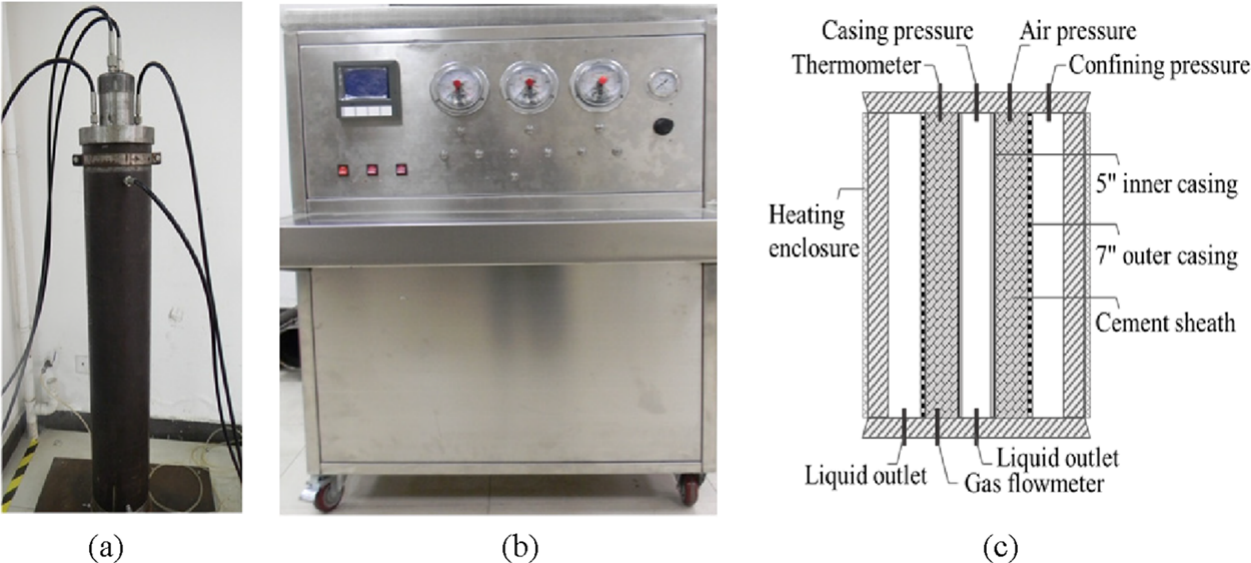
Cement sheath annular isolation simulation device designed by China Chuanqing Downhole Operation Company: (a) simulated wellbore, (b) main control box, and (c) structure diagram.

The device used in this research is the cement sheath annulus isolation simulation device designed by China Chuanqing Downhole Operation Company, as shown in Figure 1. The simulated wellbore is composed of 5″ inner casing, 7″ outer casing, upper and lower sealing caps, and pressurizing components. Injecting cement slurry between the two layers of casing can form a cement sheath. The inside of the 5″ inner casing can be pressurized and depressurized for simulating the pressure change in the downhole casing. Air pressure is applied between the 5″ inner casing and the 7″ outer casing to test the sealing ability of the cement sheath. The loading and unloading process of the cement sheath in the well is simulated by adjusting the pressure rise and fall inside the casing. During the experiment, if the gas flow rate was monitored by a gas flowmeter, it indicated that the cement sheath failed to seal, and the gas channeling path had appeared at the casing-cement sheath interface, cement sheath-formation interface, or the cement sheath body. The working pressure of the inner casing of the device can reach 70 MPa, the maximum working temperature is 90 °C, and the simulated wellbore height is 1 m.

### 2.3 Experiment Process and Results

The annular isolation simulation experiment was carried out on the typical cement Y190 and B190 mentioned above with this device. The confining pressure during the experiment was 0 MPa and did not change with the casing’s internal pressure. The process and results are explained below.

#### 2.3.1 Y190 Annular Isolation Simulation Test

The Y190 cement slurry was injected between the 5″ and 7″ casings and cured at room temperature and pressure for 7 days. Then, the following steps were carried out: (1) the casing internal pressure was raised to 40 MPa; (2) 2 MPa air pressure was applied in the cement sheath annular space to test gas channeling.

**Figure 2:**
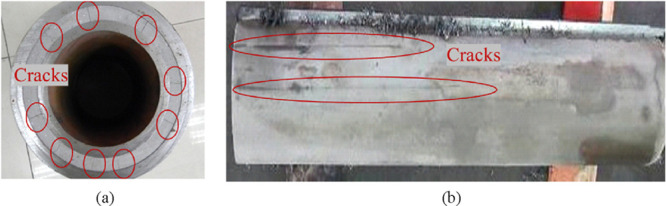
Cutting results of the Y190 cement sheath simulated wellbore: (a) transverse section view and (b) longitudinal section view.

The test phenomenon is as follows: (1) When the casing internal pressure rises to 35 MPa, a clear sound of cement ring rupture is heard; (2) After the pressure test is completed, the pressure is released, and gas channeling is found immediately after 2 MPa air pressure is applied to the annular space; there is still gas channeling when the pressure drops to 1 MPa. Then, the simulated wellbore was cut into transverse and longitudinal sections to observe the failure form of the cement sheath, as shown in Figure 2.

Figure 2a transverse section view shows that under the action of casing internal pressure, radial cracks extending from the outer wall of the 5″ casing to the inner wall of 7″ casing were generated on the circumference of the cement sheath. From the stress–strain principle of thick-walled cylinders, this is an obvious tensile crack damage (or tensile failure). What casing pressure destroys is the tensile strength, which is one of the weakest strengths of cement sheath.

Figure 2b longitudinal section view shows that this transverse radial crack extends along the axial direction and runs through the entire simulated wellbore, proving that the cement sheath body crack is the direct cause of gas channeling.

#### 2.3.2 B190 Annular Isolation Simulation Test

**Figure 3:**
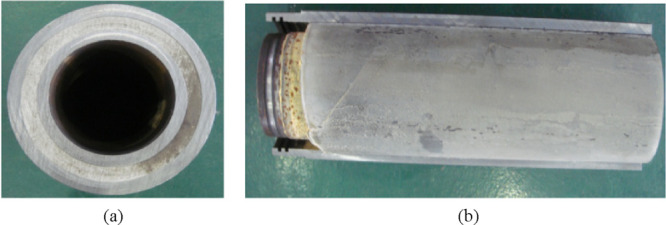
Cutting results of B190 cement sheath simulated wellbore: (a) transverse section view, and (b) longitudinal section view.

The curing method and test steps of the B190 cement sheath are the same as those of Y190. The test phenomenon is as follows: (1) When the casing internal pressure rises to 40 MPa, no obvious cracking sound is heard; (2) After the pressure was released, 2 MPa air pressure was applied in the annular space, and gas channeling occurred after waiting for 10 min. Then, the simulated wellbore was cut into transverse and longitudinal sections, as shown in Figure 3.

From the transverse and longitudinal section view, no directly observable macroscopic cracks were found. However, when 1.8 MPa air pressure was applied in the annular space, gas channeling was still found after 10 min, indicating that there was a channeling path. It is speculated that the channeling path is the micro-annulus existing in the annulus. Because B190 is a modified cement, the tensile strength is improved, and B190 undergoes plastic yield with the increase of casing internal pressure. After the pressure is released, the inner casing shrinks, and the first cemented surface cannot be recovered, forming a micro-annulus.

The B190 annulus isolation simulation test shows that the plastic yield of cement sheath is also the integrity failure form of the cement sheath.

The results obtained from the above tests are consistent with the existing knowledge about the failure form of cement sheaths. The tensile crack damage of cement sheath and the generation of micro-annulus by plastic yielding are the main behaviors of cement sheath integrity failure. However, this experiment also uses gas permeability as an evaluation variable, and there are similar problems, namely, long test periods and high cost.

Therefore, according to the experimental phenomena, we propose a cement sheath integrity failure model based on the continuum mechanics theory and establish damage variables to analyze the two main forms of cement sheath integrity failure.

## 3 Cement Sheath Mechanical Integrity Analysis Model

It is assumed that the casing is centered, the wellbore is a regular circle, and the in-situ stress is uniform. According to Saint-Marc’s^13^ initial stress state model of cement sheath and the consensus on initial stress of cement sheath by Bois,^14,15^ Bosma,^16^ etc., the initial stress state of cement sheath is set as the hydrostatic pressure state in this research. That is, when the cement sheath is formed, the outward interaction force with the formation rocks around the wellbore is the hydrostatic column pressure, and the inward interaction force with the casing’s outer wall is also the hydrostatic column pressure.

### 3.1 Interaction of Cement Sheath, Casing, and Surrounding Rock in the Initial State

**Figure 4:**
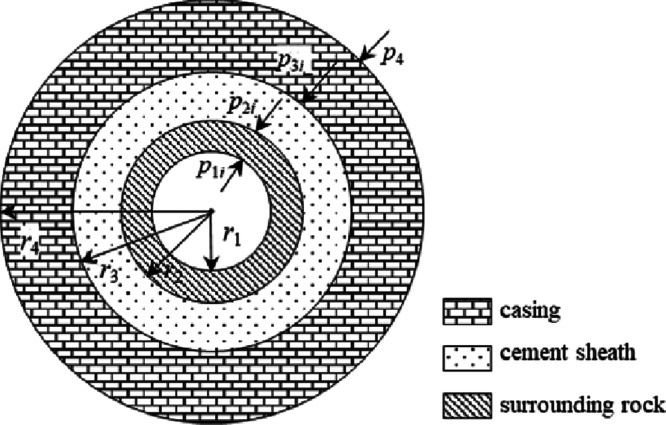
Schematic of the casing force in the initial state.

In the initial state (at the end of the setting stage), the force schematic diagram of the casing-cement sheath-surrounding rock is shown in Figure 4.

#### 3.1.1 Casing

The initial pressure of the casing’s inner wall is *p*_1*i*_, that is, the pressure of the drilling fluid column. The initial force between the casing’s outer wall and the cement sheath’s inner wall is *p*_2*i*_, that is, the hydrostatic column pressure. *r*_1_ is the casing’s inner radius and *r*_2_ is the casing’s outer radius.

According to the mechanics principle of the axisymmetric plane strain problem,^17^ the casing stress distribution is as follows:
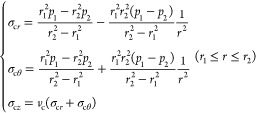
1

#### 3.1.2 Cement Sheath

The initial force between the cement sheath’s inner wall and the casing’s outer wall is *p*_2*i*_, that is, the hydrostatic column pressure. The initial force between the cement sheath’s outer wall and the surrounding rock’s inner wall is *p*_3*i*_, which is also the hydrostatic column pressure. *r*_2_ is the cement sheath’s inner radius and *r*_3_ is the cement sheath’s outer radius.

Cement sheath stress distribution:
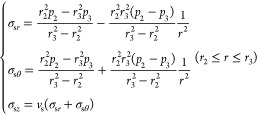
2

#### 3.1.3 Surrounding Rock

The initial force between the surrounding rock’s inner wall and the cement sheath’s outer wall is *p*_3*i*_, that is, the hydrostatic column pressure. The force on the surrounding rock’s outer wall is *p*_4_, that is, the in-situ stress of the far formation. Therefore, the value of *r*_4_ must be large enough, and it should be far away from the stress concentration area around the wellbore, so that *p*_4_ can be treated as far-ground stress. Generally, it is more than 10 times larger than the wellbore radius. In this research, *r*_4_ is taken as 100 times the wellbore radius. *r*_3_ is the wellbore radius.

Surrounding rock stress distribution:
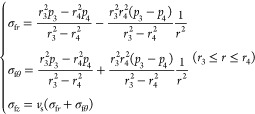
3

### 3.2 Interaction of Cement Sheath, Casing, and Surrounding Rock in Subsequent Wellbore Operations

**Figure 5:**
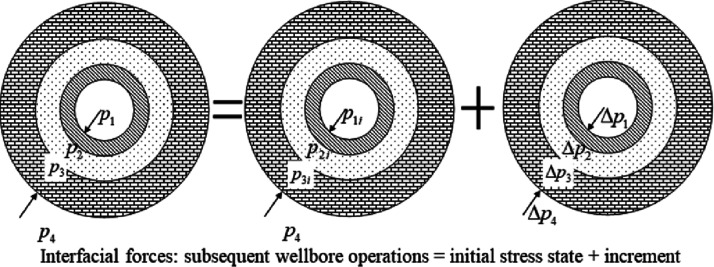
Schematic of the interfacial force superposition relationship of the casing, cement sheath, and surrounding rock during subsequent wellbore operations.

Subsequent wellbore operations, such as pressure testing, replacement of working fluid in the well, acid fracturing, and hollowing out for production, are all carried out on the initial state of the wellbore after the end of the setting stage. The casing pressure in subsequent operations can be regarded as an increment Δ*p*_1_ above the drilling fluid column pressure at the end of the settling stage. Correspondingly, the forces acting on the casing-cement sheath and cement sheath-formation bonding surfaces will generate increments Δ*p*_2_ and Δ*p*_3_.The in-situ stress will not change, its increment Δ*p*_4_ = 0, as shown in Figure 5.

The forces on the two interfaces can be decomposed as follows:
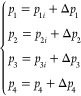
4

To get the interaction forces *p*_2_ and *p*_3_ of the cement sheath, casing and surrounding rock under subsequent wellbore operations from eq 4, the increments Δ*p*_2_ and Δ*p*_3_ must be obtained first. The casing-cement sheath-surrounding rock composite model is established for the interfacial force increment (see the right side of the equation in Figure 5). The interfacial force increment is small relative to the initial state. The deformation of casing, cement sheath, and surrounding rock is also very small, which satisfies the small deformation condition. The linear elastic constitutive model is applicable to the casing, cement sheath, and surrounding rock.

According to the mechanics principle of the axisymmetric plane strain problem under the linear elastic constitutive relation, the radial displacement formula under the interface force increment is as follows:

#### 3.2.1 Casing Radial Displacement Formula


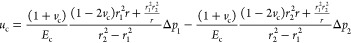
5

The radial displacement at the casing’s outer wall (*r* = *r*_2_) is
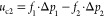
6where
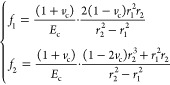


#### 3.2.2 Cement Sheath Radial Displacement Formula


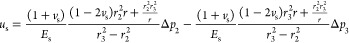
7

The radial displacement at the cement sheath’s inner wall (*r* = *r*_2_) is
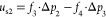
8where
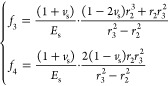


The radial displacement at the cement sheath’s outer wall (*r* = *r*_3_) is
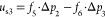
9where
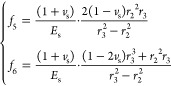


#### 3.2.3 Surrounding Rock Radial Displacement Formula


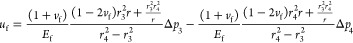
10

The radial displacement at the borehole wall (*r* = *r*_3_) is
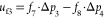
11where
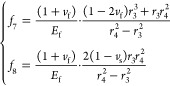


According to the radial displacement continuous condition of casing-cement sheath-surrounding rock combination, at *r* = *r*_2_ position, *u*_c2_ = *u*_s2_; at *r* = *r*_3_ position, *u*_s3_ = *u*_f3_. Therefore,
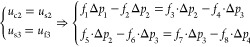
12

Solving eq 12 gives
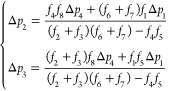
13

### 3.3 Damage Variable of Cement Sheath

**Figure 6:**
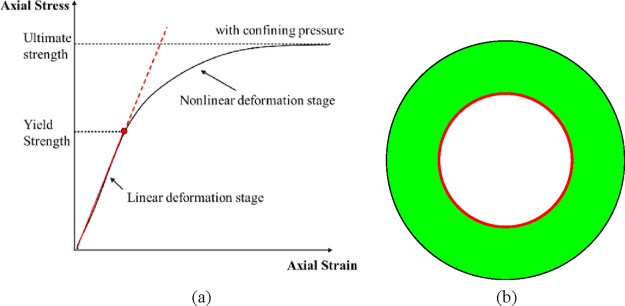
Schematic of the linear elastic constitutive equation describing cement sheath mechanical properties: (a) effective range of linear elastic constitutive equations and (b) plastic yield of the cement sheath inner wall.

The linear elastic constitutive equation has a limited effective range in describing the mechanical behavior of the cement sheath. As shown in Figure 6a, the linear elastic constitutive equation is accurate in the linear deformation stage before yield strength. However, in the nonlinear deformation stage between the yield strength and the ultimate strength, the results described by the linear elastic constitutive equation have large errors. Therefore, the linear elastic constitutive equation can be used to judge whether the cement sheath has yielded,^18^ but only for the cement sheath’s inner wall, as shown in Figure 6b. The reasons are as follows: (1) the inner wall of the cement sheath yields first, and the material properties of the inner wall deviate from the linear elastic segment after yielding. If the linear elastic constitutive equation is still used to describe the yielding inner wall of the cement sheath, it is inaccurate to calculate whether the outer cement sheath has yielded or not. (2) Similarly, when yielding further extends to the outer wall of the cement sheath, if the yielded part is still described by the linear elastic constitutive equation, it is also inaccurate to calculate whether the unyielded part yields or not. Therefore, the position where the linear elastic constitutive equation can accurately judge whether to yield or not is only the inner wall of the cement sheath.

Only the plastic yielding of the cement sheath inner wall is not enough to indicate that the mechanical integrity of the cement sheath has been destroyed. This should be demonstrated based on whether most of the cement sheath’s material functions are intact. In this research, the damage variables in the continuum mechanics theory are used to describe the failure of the cement sheath material function and to measure the mechanical integrity of the cement sheath.

In terms of the microstructure, there are defects inside the material, such as microvoids and microcracks. The mechanical properties and stress–strain response of most engineering materials are largely attributed to microdefects within the material. There are also microdefects in concrete and cement stone. From a microscopic analysis, the nonlinear stress–strain characteristics of concrete and cement stone are mainly due to the generation and aggregation of microcracks. The generation and accumulation of microcracks will gradually reduce the strength and reduce the bearing capacity. Ultimately, the microcracks aggregate into large cracks, which penetrate through the concrete or cement stone to break and shatter, and the strength is completely lost. This is a process of gradual deterioration of material properties, and the degree of deterioration can be described by the damage variable of the continuum damage theory.^19^ The continuum damage theory^20^ holds that the response of the material depends only on the current state of the microstructural arrangement, which can be described by a set of internal variables, which are called damage variables.

**Figure 7:**
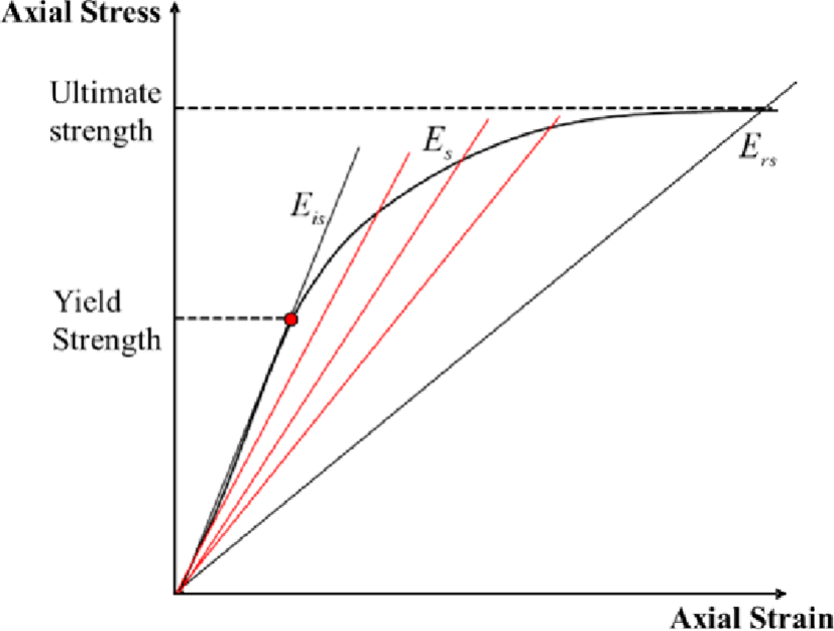
Schematic of the damage process of cement sheath stiffness.

As shown in Figure 7, taking the cement sheath as an example, *E*_is_ is defined as the Young’s modulus of the initial linear stage, *E*_s_ is Young’s modulus after the cement sheath stress exceeds the yield strength, and *E*_rs_ is the residual Young’s modulus at which the cement sheath stress reaches the ultimate strength (fractured or crushed). When the stress is lower than the yield strength, the deformation of the cement sheath is in the linear elastic stage. The cement sheath is not damaged, the material function is intact, and the mechanical integrity is maintained, so the damage variable *d* is 0.

On the contrary, when the stress reaches or exceeds the ultimate strength, the cement sheath begins to crack or even crush. The material function of the cement sheath is damaged to a certain stage, and it can still bear the residual load, but the mechanical integrity of the cement sheath has been completely lost, so the damage variable *d* is 1.

Most importantly, when 0 < *d* < 1, the cement sheath is in the nonlinear deformation stage and the stiffness (Young’s modulus) of the cement sheath begins to decrease. The graph shows that the slope of the red line begins to decrease. This indicates that the material function of the cement sheath is damaged and the mechanical integrity is gradually lost. The microscopic manifestation is that microcracks begin to generate and aggregate. Therefore, the damage process can be described in terms of the reduction rate of Young’s modulus.

Therefore, the damage variable of the cement sheath mechanical integrity is defined as follows:
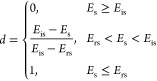
14where *d* is the damage variable of the cement sheath mechanical integrity, *E*_s_ is the Young’s modulus in the damage stage, *E*_is_ is the undamaged Young’s modulus, and *E*_rs_ is the residual Young’s modulus. The three Young’s moduli are all secant moduli, as shown in Figure 7. It can be seen that the mechanical integrity of the cement sheath can be quantitatively described by the damage variable *d*.

The initial Young’s modulus *E*_is_ and residual Young’s modulus *E*_rs_ used in solving the equation of *d* can be determined by mechanical tests of cement sheath. Young’s modulus *E*_s_ in the damage stage is the variable Young’s modulus of the cement sheath in the nonlinear deformation stage, which can be determined by the nonlinear elastic description equation. See Appendix for the solution model and calculation formula.

### 3.4 Forms of Cement Sheath Integrity Failure

According to the simulation experiment of cement sheath annulus isolation, the main failure forms of cement sheath are tensile crack damage and micro-annulus caused by plastic yielding, and the criteria for judging these two failure forms are the maximum tensile stress criterion and the Mohr–Coulomb criterion.^21−23^ Therefore, the mechanical integrity analysis model of the cement sheath uses the linear elastic constitutive equation to calculate the stress distribution of the cement sheath and qualitatively judges the mechanical integrity of the cement sheath according to the maximum tensile stress criterion and the Mohr–Coulomb criterion. Finally, the damage variable *d* is used to quantitatively judge the mechanical integrity of the cement sheath.

**Figure 8:**
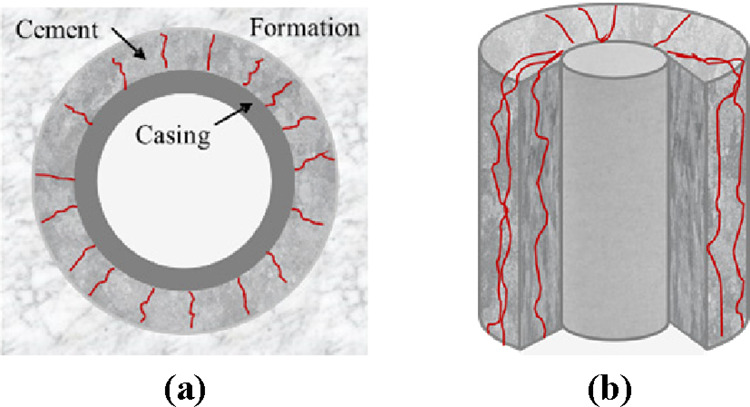
Schematic of cement sheath tensile crack damage: (a) cross section and (b) longitudinal section.

**Figure 9:**
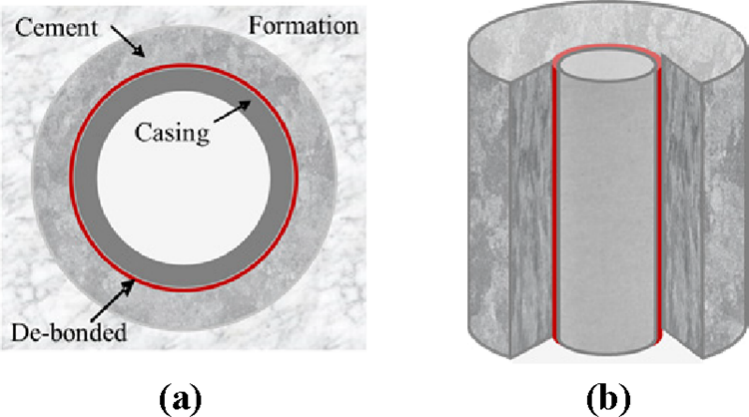
Schematic of casing debonding or shrinking micro-annulus: (a) cross section and (b) longitudinal section.

**Figure 10:**
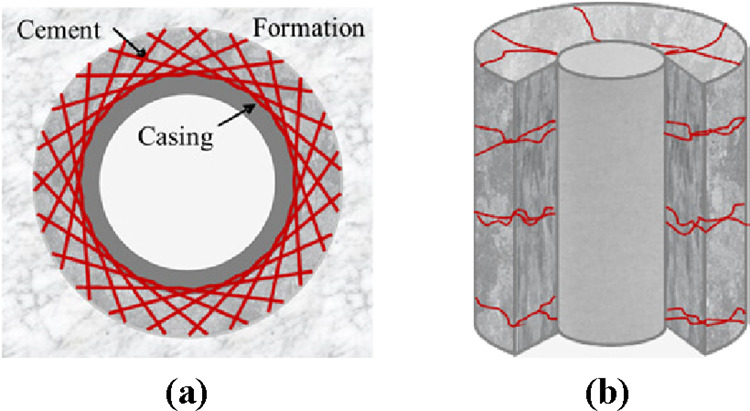
Schematic of cement sheath damage when 0 < *d* ≤ 1: (a) cross section and (b) longitudinal section.

According to the qualitative and quantitative judgment methods of the cement sheath mechanical integrity analysis model, the interpretations^13,24^ of the calculation results are determined as follows:

(1) When the stress calculated by the model meets the maximum tensile stress criterion, that is, one of the three principal stresses in the cement sheath, the radial principal stress, the circumferential principal stress, or the axial principal stress, is greater than the tensile strength of the cement sheath. The cement sheath will be damaged by tensile cracks. Interpret this situation as tensile crack, as shown in Figure 8.
(2) When the radial stress in the model calculation results is tensile stress on the first cementation surface, the casing and cement sheath will be debonding due to the low transverse bonding strength of the cement sheath (usually 2–3 MPa). Interpret this situation as casing debonding micro-annulus, as shown in Figure 9.
(3) When the calculated stress results of the model satisfy the Mohr–Coulomb criterion, the cement sheath will yield plastically. If the casing’s internal pressure decreases, the casing will shrink and a micro-annulus will be created on the first cementation surface. Because of the premise of casing shrinkage, this situation is interpreted as casing shrinking micro-annulus, as shown in Figure 9.
(4) The mechanical integrity of the cement sheath is quantitatively described by the damage variable *d*, as shown in Figure 10. When 0 < *d* < 1, the cement sheath has different degrees of damage, the greater the value of *d*, the higher the degree of damage. When *d* = 1, the cement sheath has been cracked or even crushed, the mechanical integrity is completely lost.

## 4 Results and Discussion

### 4.1 Parameters of the Cement Sheath Mechanical Integrity Analysis Model

Take the Longgang X well in the Longgang gas field in Sichuan as an example. The cement sheath of 127 mm oil layer casing at the depth of 6000 m well was selected to analyze its mechanical integrity under different downhole conditions.

The cement slurry system for 127 mm oil layer casing cementing in Longgang X well is Grade G cement + high temperature stabilizer + micro silica fume + 3% SDP-1 + 1.5% SD66 + 2.7% FS-31L + 5% SD10 + 0.44 W/C. The cement slurry was prepared in the laboratory for mechanical testing, and the cement and water used were those taken on-site during the cementing of Well X.

According to the standard GB19139 “Test Method for Oil Well Cement”, the maximum pressure for cement stone curing is 20.7 MPa. At present, the optimal pressure set by the curing kettle is also 20.7 MPa, which cannot simulate the hydrostatic pressure of 60 MPa. The curing pressure of the cement stone has not reached 60 MPa, and it is not suitable to add 60 MPa confining pressure for the triaxial stress test of the cement stone. Therefore, the maximum pressure of 20.7 MPa specified by the standard is adopted as the curing pressure of the cement sheath and the confining pressure of the triaxial stress test.

According to the determined pressure and downhole measured temperature, set the curing conditions of cement stone: 130 °C × 21 MPa × 7 days.

The conditions of the triaxial stress test are 130 °C × 20 MPa and 130 °C × 10 MPa, and the confining pressure is changed to measure the cohesion and internal friction angle of the cement stone.

Curing conditions for the tensile strength test: 130 °C × 21 MPa × 7 days.

The main input parameters of the cement sheath mechanical integrity analysis model are tensile strength, cohesion, internal friction angle, Young’s modulus, Poisson’s ratio, fitted bulk modulus, and shear modulus of the cement sheath; yield strength, Young’s modulus, and Poisson’s ratio of the casing; Young’s modulus and Poisson’s ratio of the formation. The geometric parameters are the inner and outer diameter of the casing and the radius of the wellbore. These parameters are divided into experimental measurement data and fitting parameters, as described below.

#### 4.1.1 Experimental Measurement Data

**Figure 11:**
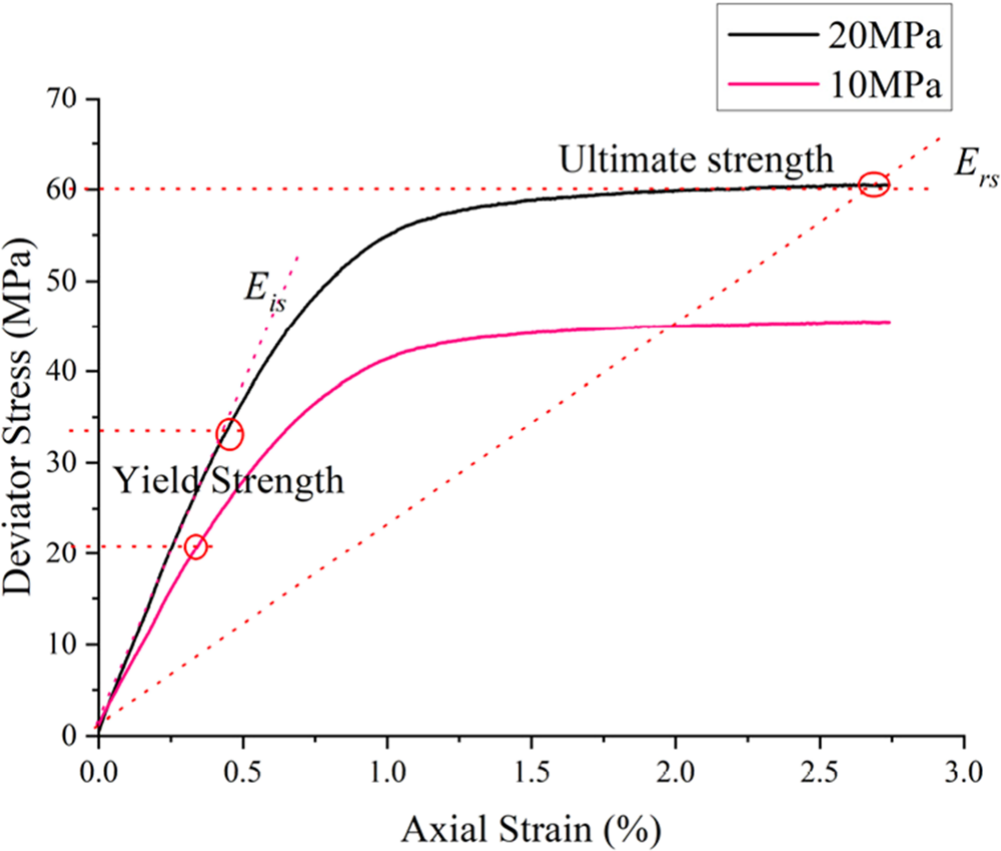
Triaxial stress–strain curves of cement stone under different confining pressures.

The triaxial stress–strain curves of cement stone samples under 10 and 20 MPa confining pressure are shown in Figure 11. The strength parameters under different confining pressures are read from the curves, and the calculation method^19^ given in the literature^17^ is used to obtain the cohesive force and internal friction angle of the cement stone, as shown in Table 2.

**Table 2:** Strength Parameters, Cohesion, and Internal Friction Angle of Cement Stone

	yield strength (MPa)		calculation results	
confining pressure (MPa)	differential stress	axial stress	cohesion (MPa)	internal friction angle (deg)
10	21	31	0.415	35.74
20	34	54		

Based on the stress–strain curve of the cement stone under 20 MPa confining pressure (as shown in Figure 11), the initial Young’s modulus, Poisson’s ratio, and residual Young’s modulus under the ultimate load of the linear segment were calculated. The tensile strength is determined according to GB19139 “Test Method for Oil Well Cement”. Young’s modulus, Poisson’s ratio, and tensile strength are shown in Table 3.

**Table 3:** Mechanical Parameters of Cement Stone

initial Young’s modulus (MPa)	initial Poisson’s ratio	residual Young’s modulus (MPa)	tensile strength (MPa)
7653.30	0.092	2238.94	2.3

#### 4.1.2 Fitting Parameters

**Figure 12:**
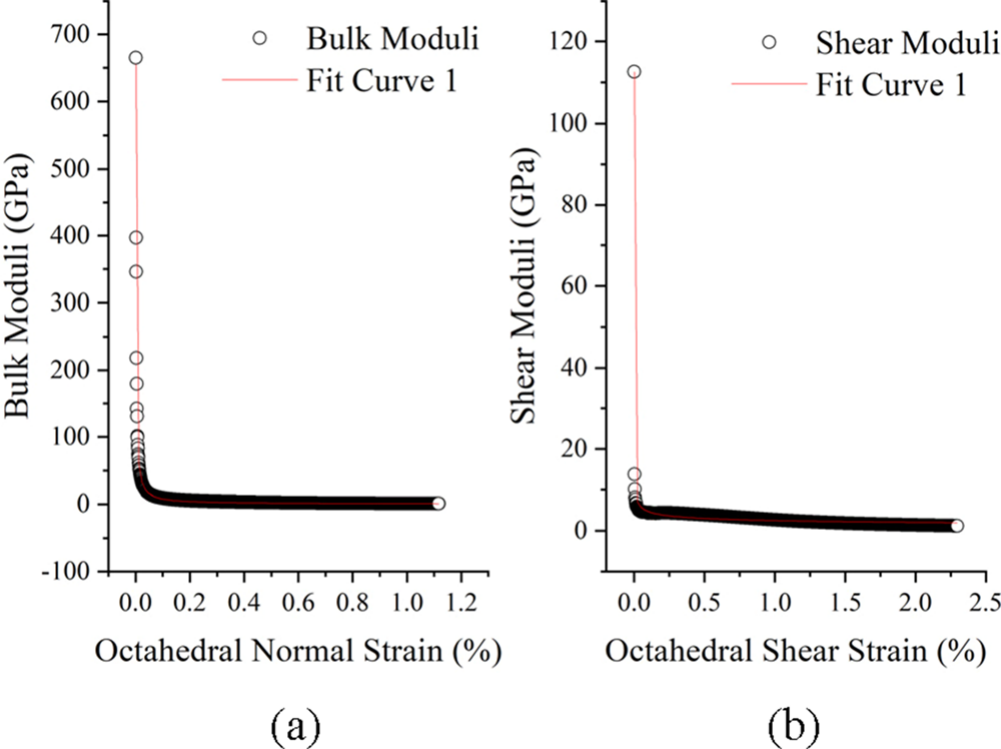
Function fitting of cement stone (a) bulk modulus and (b) shear modulus.

The stress–strain curves of the triaxial stress test at a confining pressure of 20 MPa and a temperature of 130 °C were fitted with the bulk modulus and shear modulus functions. The bulk modulus fitting function form is , and the shear modulus fitting function form is *G*_s_ = *b*(γ_oct_ – *c*)^*n*^. The fitting graph is shown in Figure 12, and the coefficient fitting results and fitting effect quantification parameters (reduced Chi-square and adjusted R-square) are shown in Table 4. It can be seen from the two quantification parameters that the fitting effect is quite good.

**Table 4:** Fitting Results of Cement Stone Bulk Modulus and Shear Modulus

Bulk Modulus: *K*_s_ = *a*ε_oct_^*m*^			
fit coefficients			
*a*	*m*	reduced χ^2^	adjusted *R*^2^
0.88398	–0.95891	0.96701	0.99853

Shear Modulus: *G*_s_ = *b*(γ_oct_ – *c*)^*n*^				
fit coefficients				
*b*	*c*	*n*	reduced χ^2^	adjusted *R*^2^
2.49435	2.155 × 10^–4^	–0.27723	0.36249	0.96404

According to the cementing design of 127 mm oil layer casing in the Longgang X well, the parameters of casing and formation (the rock is limestone) can be obtained; see Table 5 for details.

**Table 5:** Physical Parameters of Casing and Formation

parameter	value	parameter	value
casing inner diameter	54.31 mm	casing steel grade	TP110TS
casing outer diameter	63.5 mm	casing Young’s modulus	206.85 × 103 MPa
wellbore radius	82.0 mm	casing Poisson’s ratio	0.3
casing yield strength	758.3 MPa	formation Young’s modulus	54.12 × 103 MPa
formation Poisson’s ratio	0.218		

### 4.2 Cement Sheath Mechanical Integrity Analysis under Different Conditions

From the end of the setting stage to the production stage, the wellbore operating conditions mainly include the initial state, pressure test, acid fracturing, clear water kill, and casing hollowing out. Taking the initial state as the starting point, all the working conditions are regarded as independent, and the cement sheath mechanical integrity under these working conditions is analyzed separately. In this way, it is determined under which operating conditions the cement sheath mechanical integrity faces the greatest risk, and the damage degree is quantified.

#### 4.2.1 In the Initial State

In the setting stage, the drilling fluid density is 1.3 g/cm^3^, and the pressure at the 6000 m downhole is about 76.5 MPa. In the initial state at the end of the setting stage, the inner pressure of the casing at 6000 m is 76.5 MPa, and the hydrostatic column pressure is about 60 MPa on the inner and outer cementation surfaces of the cement sheath (the subsequent operating conditions are the same).

##### 4.2.1.1 Cement Sheath Tensile Crack

**Figure 13:**
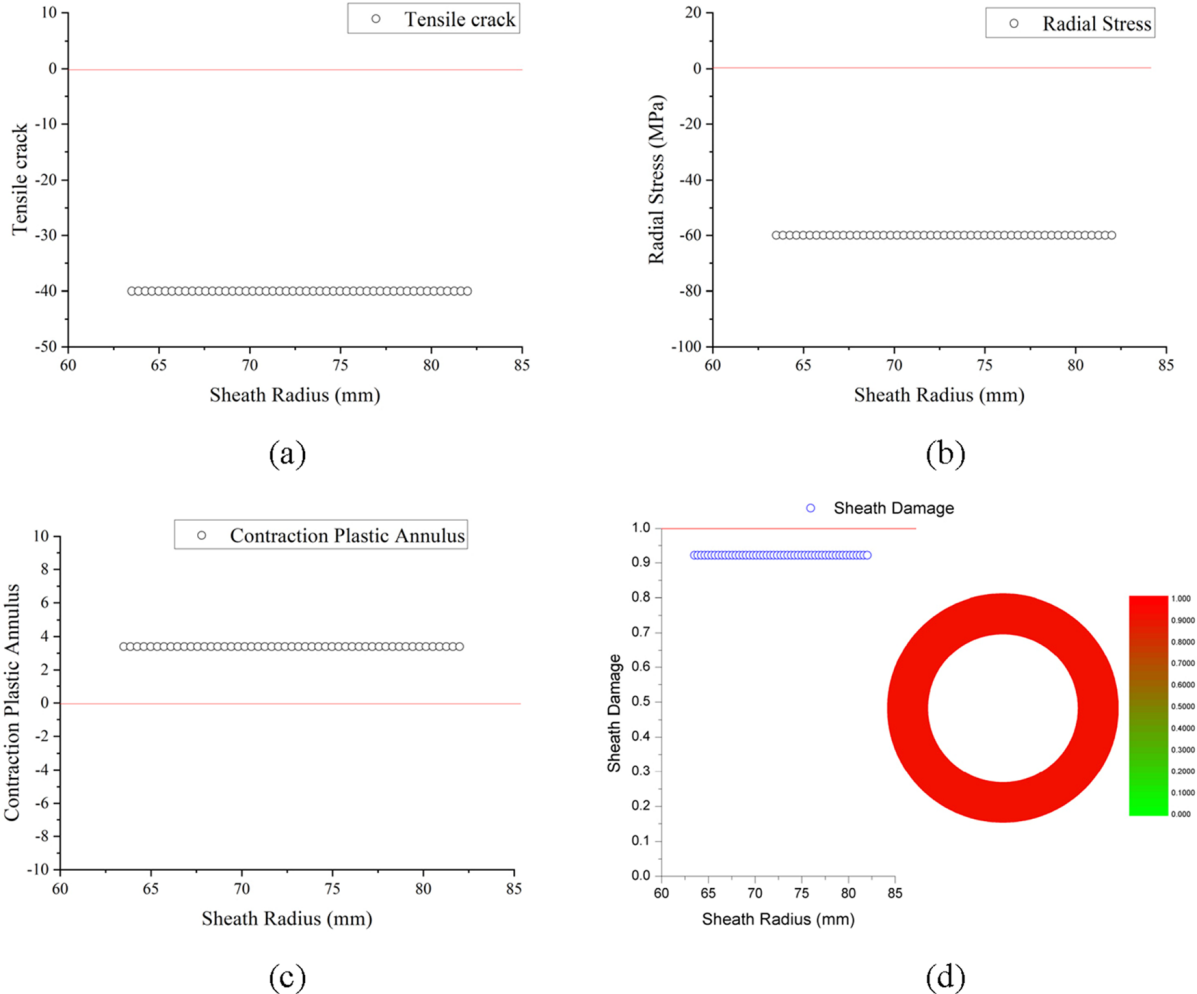
Calculation results in the initial state: (a) cement sheath tensile cracks, (b) casing debonding micro-annulus, (c) casing shrinking micro-annulus, and (d) cement sheath damage variable.

The above Figure 13a shows that the check value of the maximum tensile stress criterion of the cement sheath in the initial state calculated by the cement sheath mechanical integrity analysis model is all less than 0, and it is judged that there will be no tensile cracks in the initial state.

##### 4.2.1.2 Casing Debonding Micro-Annulus

The above Figure 13b shows that the radial stress of the cement sheath in the initial state is all less than 0, which is all compressive stress. It is judged that there will be no casing debonding micro-annulus in the initial state.

##### 4.2.1.3 Casing Shrinking Micro-Annulus

The above Figure 13c shows that the Mohr–Coulomb criterion check value of the cement sheath in the initial state is all greater than 0, indicating that the cement sheath has undergone plastic yielding, and if the casing shrinks, a micro-annulus will appear on the casing-cement sheath interface. It is judged that the casing shrinkage micro-annulus will appear in the initial state.

##### 4.2.1.4 Damage Variable of Cement Sheath Mechanical Integrity

The above Figure 13d shows that the damage variable of the cement sheath mechanical integrity in the initial state calculated by the cement sheath mechanical integrity analysis model has reached more than 0.9. The cement sheath is on the verge of breaking, and the mechanical integrity has lost 90%. The calculated results are in good agreement with the phenomena observed in Jackson and Murphey’s^8^ cement sheath annulus isolation simulation experiments. (The inner casing was pressurized at 10,000 psi, and the outer annulus was pressurized at 1000 psi. After curing, gas channeling occurred just after the application of air pressure in the annular space. No matter whether the pressure of the inner casing rises or falls, there was always gas channeling in the whole test process.)

The mechanical integrity damage variable of the cement sheath quantitatively reveals that the mechanical properties of the cement sheath at the end of the setting stage can no longer meet the requirements of the downhole pressure environment. The calculation results also show that the damage degrees of the inner and outer walls of the cement sheath are almost the same, indicating that the imagined situation of large differences in the damage between the inner and outer walls of the cement sheath does not exist. After the inner wall of the cement sheath is damaged, it cannot be expected to rely on the outer wall to maintain the sealing performance. The cement sheath is too thin to show damage differences in the mechanical integrity of the inner and outer walls.

#### 4.2.2 In Pressure Test Stage

In the pressure test stage, the wellhead pressure is 50 and 60 MPa, the working fluid is clear water with a density of 1.0 g/cm^3^, and the pressure at 6000 m downhole is about 110 and 120 MPa, respectively. Taking the wellhead pressure test of 50 MPa as an example, the mechanical integrity of the cement sheath is analyzed.

##### 4.2.2.1 Cement Sheath Tensile Crack

**Figure 14:**
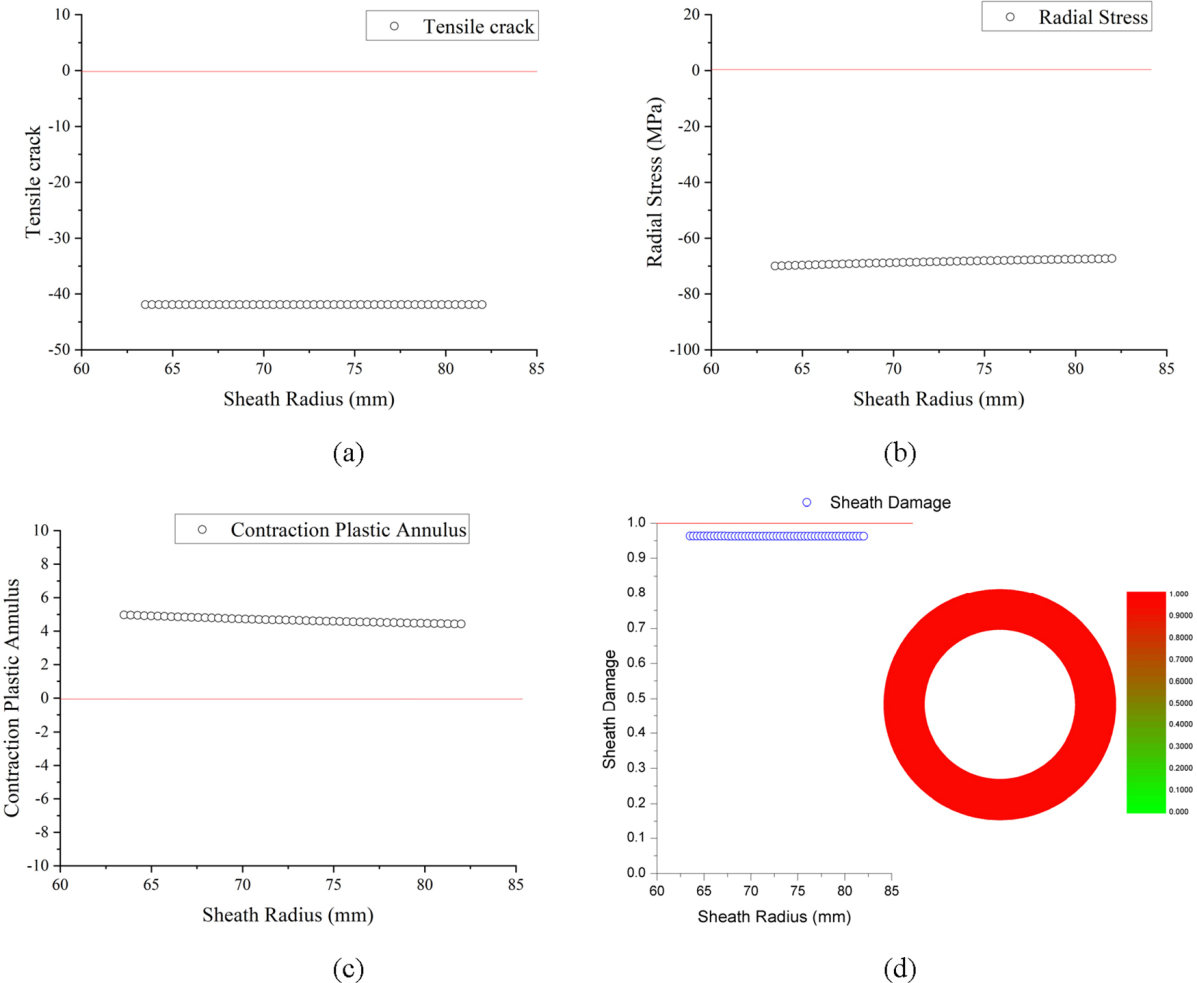
Calculation results in the pressure test stage (50 MPa): (a) cement sheath tensile cracks, (b) casing debonding micro-annulus, (c) casing shrinking micro-annulus, and (d) cement sheath damage variable.

The above Figure 14a shows that the check value of the maximum tensile stress criterion of the cement sheath in the pressure test stage (50 MPa) is all less than 0, and it is judged that there will be no tensile cracks in this stage.

##### 4.2.2.2 Casing Debonding Micro-Annulus

The above Figure 14b shows that the radial stress of the cement sheath in the pressure test stage (50 MPa) is all less than 0, which is all compressive stress. It is judged that there will be no casing debonding micro-annulus.

##### 4.2.2.3 Casing Shrinking Micro-Annulus

The above Figure 14c shows that the Mohr–Coulomb criterion check value of the cement sheath in the pressure test stage (50 MPa) is all greater than 0, indicating that the cement sheath has undergone plastic yielding, and if the casing shrinks, a micro-annulus will appear on the casing-cement sheath interface. It is judged that the casing shrinking micro-annulus will appear in the pressure test stage (50 MPa).

##### 4.2.2.4 Damage Variable of Cement Sheath Mechanical Integrity

The above Figure 14d shows that the damage variable of the cement sheath mechanical integrity in the pressure test stage (50 MPa) has reached about 0.95. The mechanical integrity of the cement sheath has lost 95%.

#### 4.2.3 Summary of Different Operating Conditions

According to the cement sheath mechanical integrity analysis model, the sealing failure at 6000 m underground in Longgang X well was analyzed. The results are summarized in Table 6.

**Table 6:**
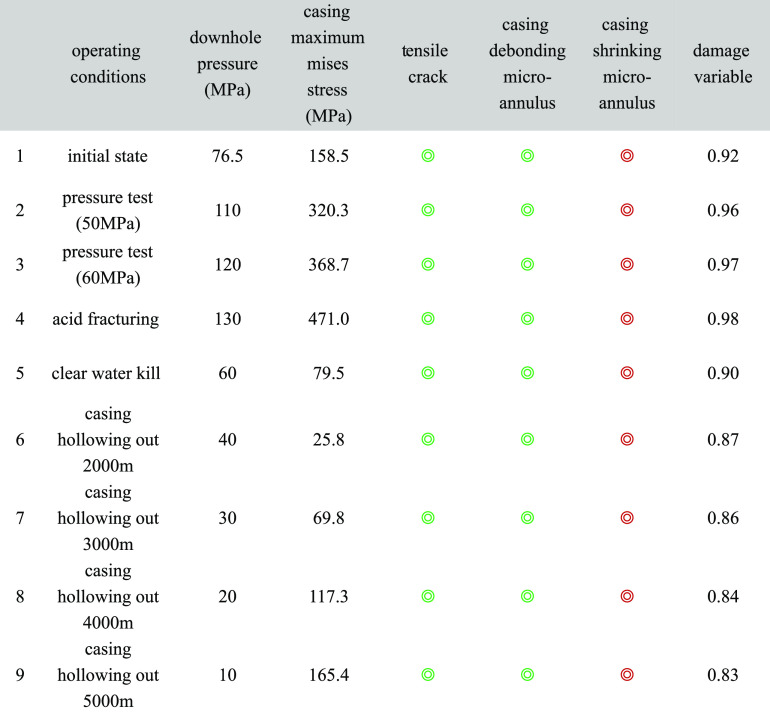
Summary of Cement Sheath Mechanical Integrity Analysis under Different Operating Conditions^a^

a green circles mean it will not happen; red circles mean it will happen.

It can be seen from the results in Table 6 that (1) according to the qualitative understanding of the linear elastic constitutive equation, from the initial state to the hollowing out 5000 m, 9 operating conditions, the calculation analysis shows that the cement sheath will not have tensile cracks and casing debonding micro-annulus. However, casing shrinking micro-annulus occurs in every operating condition. (2) According to the quantitative description of the nonlinear elastic constitutive equation, the mechanical integrity damage variable of cement sheath under 9 working conditions is calculated. The lowest damage variable was found to be 0.83 and the highest was 0.98. This means that at least 83% of the mechanical integrity of the cement sheath in downhole operations is lost, and the annular isolation effect cannot be guaranteed.

**Figure 15:**
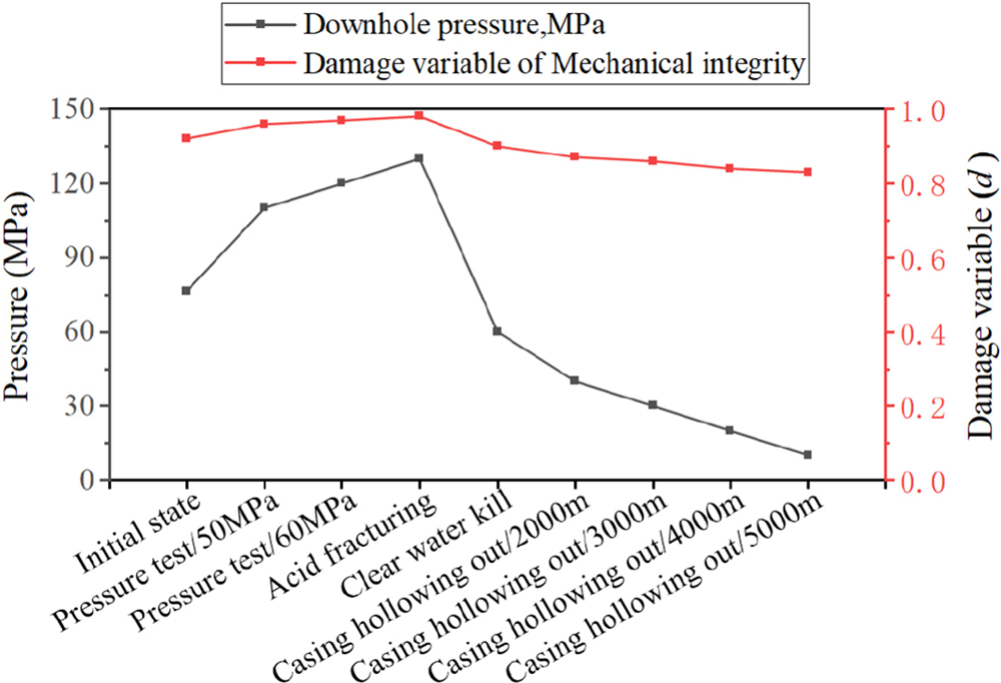
Evolution trend of the cement sheath mechanical integrity damage variable with downhole pressure.

It can be seen from Figure 15 that the cement sheath mechanical integrity damage variable is closely related to downhole pressure. As the downhole pressure increases, so does the risk of mechanical integrity damage. This conclusion is consistent with the results of the cement sheath annulus isolation simulation experiments done by Goodwin and Crook,^7^ Jackson, and Murphe.^8^ Their experiments found that with the increase of casing pressure, the damage to the cement sheath increases, and the pressure in the casing below 2000 psi does not cause gas channeling; while the pressure in the casing is higher than 4000 psi, the gas channeling is very obvious.

Therefore, the cement sheath mechanical integrity analysis model found that the mechanical properties of the Longgang X well cement slurry system could not meet the operating requirements at 6000 m underground. Reducing wellbore pressure will help maintain the mechanical integrity of the cement sheath, providing sealing performance.

## 5 Conclusions

Based on the analysis of the interaction relationship between the cement sheath interfaces, the cement sheath mechanical integrity analysis model is established, and the application of the model is tested with field examples. The main findings are as follows:

(1) Through the simulation experiment of cement sheath annulus isolation, the main form of cement sheath integrity failure was obtained, that is, tensile crack damage and micro-annulus caused by plastic yielding.
(2) A damage variable of cement sheath mechanical integrity based on the theory of continuum mechanics is proposed, which can quantitatively judge the cement sheath mechanical integrity.
(3) Considering operating conditions faced by the cement sheath at oil layer casing in a well, the main failure form of cement sheath mechanical integrity is casing shrinking micro-annulus. The damage variable is closely related to downhole pressure.

## Figures and Tables

**Figure 16:**
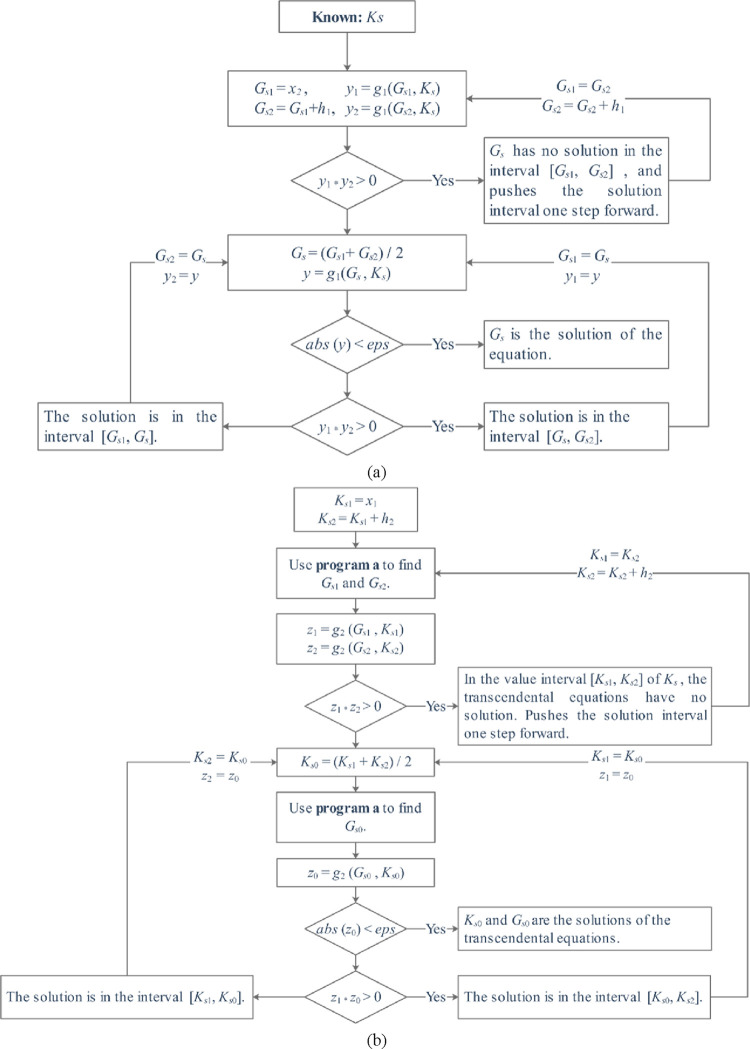
Program diagram of solving transcendental equations by dichotomy: (a) Dichotomy module that given *K*_s_ to find the *G*_s_ satisfies *g*_1_(*G*_s_, *K*_s_) = 0, and (b) program diagram of the bulk modulus and shear modulus transcendental equation solution process.

## Appendix: Solving Transcendental Equations By The Dichotomy

Andenaes, Cedolin, Kupfer, and Gerstle^19^ proposed that under the action of confining pressure, there is a unified functional relationship between the octahedral normal stress and the octahedral normal strain before the ultimate load or peak stress of different concrete. In addition, there is also a unified functional relationship between the octahedral shear stress and the octahedral shear strain. According to previous studies, cement stone is the same as concrete, and there is a unified functional relationship between the secant bulk modulus and octahedral normal strain and between the secant shear modulus and octahedral shear strain of different cement stones.

The functional form of the bulk modulus is

15

where *K*_s_ is the bulk modulus, GPa; ε_oct_ is the octahedral normal strain, %; and *a* and *m* are the fitted material parameters.

The functional form of the shear modulus is

16

where *G*_s_ is the shear modulus, GPa; γ_oct_ is the octahedral shear strain, %; and *b*, *c*, and *n* are the fitted material parameters.

The bulk modulus and shear modulus can be converted to Young’s modulus and Poisson’s ratio using eq 17:

17

For an axisymmetric cement sheath, the radial stress σ_*r*_, circumferential stress σ_θ_, and axial stress σ_*z*_ are the three principal stresses, and the radial strain ε_*r*_, circumferential strain ε_θ_, and axial strain ε_*z*_ are the three principal strains. The description equations that express the nonlinear deformation of cement stone by the principal stresses and principal strains are as follows:

18

Let  and . Then:

19

From σ_*z*_ = *v*_s_(σ_*r*_ + σ_*θ*_) and , with *C* defined as , the following expression can be obtained:

20

From eqs 19 and 20, we get:

21

According to the units of the fitted data, the units of *A*, *B*, and *C* are GPa, the units of ε_*r*_, ε_θ_, and ε_*θ*_ are %, and the sign is negative (the tensile stress is positive), and the units of σ_*r*_, σ_θ_, and σ_*z*_ are MPa. Equation 21 also has unit conversion coefficients, such as

22

The same can be obtained:

23

24

Addition of eqs 22–24 gives:

25

Subtracting eq 23 from eq 22 gives:

26

Subtracting eq 23 from eq 24 gives:

27

Subtracting eq 22 from eq 24 gives:

28

From  and , it follows that:

29

30

From *K*_s_ = *a*ε_oct_^*m*^ and *G*_s_ = *b*(γ_oct_ – *c*)^*n*^, it follows that:

31

32

Substituting eqs 31 and 32 into eqs 29 and 30, we can get

33

where , .

Equation 33 is a transcendental equation with two variables, which can be solved by the dichotomy method.

Let the first formula of eq 33 be a function:

34

Let the second formula of eq 33 be a function:

35

The steps to solve the transcendental equations with two variables are as follows (see Figure 16 for the detailed process):

(1) Let *K*_s1_ = *x*_1_. Then eqs 24 and 35 become *g*_1_(*G*_s_, *K*_s1_) = 0 and *g*_2_(*G*_s_, *K*_s1_) = 0.
(2) Find *G*_s_ that satisfies *g*_1_(*G*_s_, *K*_s1_) = 0 by dichotomy. Let *G*_s1_ = *x*_2_ and *y*_1_ = *g*_1_(*G*_s1_, *K*_s1_); let *G*_s2_ = *G*_s1_ + *h*_1_ and *y*_2_ = *g*_1_(*G*_s2_, *K*_s1_). Determine whether *y*_1_·*y*_2_ is less than zero. If it is less than zero, it means that in the value interval [*G*_s1_, *G*_s2_] of *G*_s_, there is a solution for *g*_1_(*G*_s_, *K*_s1_) = 0. The dichotomy can be used to solve in this interval. If it is greater than zero, it means that the equation system has no solution in the value interval [*G*_s1_, *G*_s2_] of *G*_s1_; then, upon pushing the value interval of *G*_s_ one step forward (*G*_s1_ = *G*_s2_ and *G*_s2_ = *G*_s2_ + *h*_1_). A range of intervals that has a solution should be found. *G*_s_ should be obtained, *g*_2_(*G*_s_, *K*_s1_) should be brought in, and *z*_1_ = *g*_2_(*G*_s_, *K*_s1_) can be obtained.
(3) Let *K*_s2_ = *K*_s1_ + *h*_2_. Then eqs 24 and 35 become *g*_1_(*G*_s_, *K*_s2_) = 0 and *g*_2_(*G*_s_, *K*_s2_) = 0. Similarly, the dichotomy method can be used to find *G*_s_ that satisfies *g*_1_(*G*_s_, *K*_s2_) = 0, and then *g*_2_(*G*_s_, *K*_s2_) should be brought in to get *z*_2_ = *g*_2_(*G*_s_, *K*_s2_).
(4) Whether *z*_1_·*z*_2_ is less than zero should be determined. If it is less than zero, it means that in the value interval [*K*_s1_, *K*_s2_] of *K*_s_ there is a solution for the transcendental equations. The dichotomy can be used to solve in this interval. If it is greater than zero, it means that the equation system has no solution in the value interval [*K*_s1_, *K*_s2_] of *K*_s_, so the value interval of *K*_s_ is pushed one step forward (*K*_s1_ = *K*_s2_ and *K*_s2_ = *K*_s2_ + *h*_2_). A range of intervals that has a solution should be found.

By solving the transcendental eq 33 by the dichotomy method (Figure 16), the bulk modulus *K*_s_ and shear modulus *G*_s_ corresponding to the given radial stress σ_*r*_ and circumferential stress σ_θ_ of the cement sheath can be obtained. The bulk modulus and shear modulus can be converted to Young’s modulus and Poisson’s ratio. The radial stress σ_*r*_ and circumferential stress σ_θ_ of the plane strain problem have no relationship with the material parameters of the cement sheath but are only related to the internal and external pressure and geometric parameters. The radial stress σ_*r*_ and the circumferential stress σ_θ_ are directly determined by the cement sheath stress distribution formula. Therefore, after determining the force acting on the cement sheath, Young’s modulus and Poisson’s ratio distribution in the cement sheath under the internal and external loads can be determined.
